# Un carcinome épidermoide du cuir chevelu

**DOI:** 10.11604/pamj.2013.15.112.2963

**Published:** 2013-07-28

**Authors:** Amal Akazane, Badreddine Hassam

**Affiliations:** 1 Service de dermatologie-vénérologie CHU Ibn Sina, Maroc; 2 Faculté de médecine et de pharmacie Med V Souissi Rabat, Maroc

**Keywords:** Carcinome épidermoide, cancers cutanés, radiothérapie

## Image en médecine

Les carcinomes épidermoides (CE) sont les cancers cutanés les plus fréquents après les basocellulaires. Ils touchent les sujets à partir de 60 ans. Les principales lésions cutanées pré-cancéreuses sont: les kératoses actiniques, les cicatrices de brûlures, traumatismes. Le CE évolue sous une forme ulcéro-bourgeonnante ou superficielle. L’évolution se fait de proche en proche ou par voie hématogène. La prise en charge thérapeutique consiste en l'exérèse complète de la tumeur avec marges d'exérèse sup à 6mm, et éventuellement un curage ganglionnaire. La radiothérapie est réservée aux formes étendues. La prévention repose essentiellement sur l’éviction des agents carcinogènes et le traitement des lésions pré-carcinomateuses. Nous rapportons le cas d'un patient de 70 ans, tabagique chronique, consulte pour une tumeur occipitale évoluant depuis 3 ans, apparue sur une cicatrice d'un traumatisme antérieur. Une biopsie-exérèse de la tumeur a été réalisée, dont l'anatomopathologie objectivait un carcinome épidermoïde. Après bilan d'extension sans anomalies, le patient a été adressé pour radiothérapie.

**Figure 1:**
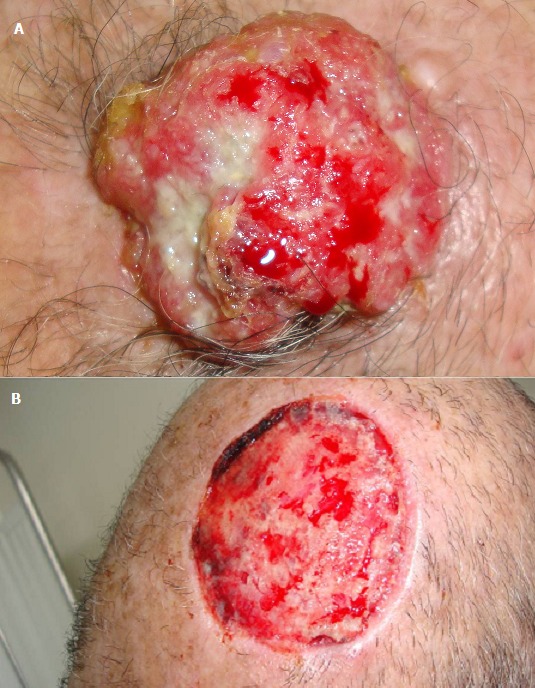
A) Tumeur ulcéro-bourgeonnante du cuir chevelu; B) Dix jours post exérèse complète avec cicatrisation dirigée

